# Cell–cell signaling in blood vessel development and function

**DOI:** 10.15252/emmm.201708610

**Published:** 2018-01-23

**Authors:** Christer Betsholtz

**Affiliations:** ^1^ Karolinska Institutet/AstraZeneca Integrated Cardio Metabolic Centre (KI/AZ ICMC) Huddinge Sweden; ^2^ Rudbeck Laboratory Department of Immunology, Genetics and Pathology Uppsala University Uppsala Sweden

**Keywords:** Cardiovascular System, Vascular Biology & Angiogenesis

## Abstract

The blood vasculature is an organ pervading all other organs (almost). During vascular development, cell–cell signaling by extracellular ligands and cell surface receptors ensure that new vessels sprout into non‐vascularized regions and simultaneously acquire organ‐specific specializations and adaptations that match the local physiological needs. The vessels thereby specialize in their permeability, molecular transport between blood and tissue, and ability to regulate blood flow on demand. Over the past decades, we have learnt about the generic cell–cell signaling mechanisms governing angiogenic sprouting, mural cell recruitment, and vascular remodeling, and we have obtained the first insights into signals that induce and maintain vascular organotypicity. However, intra‐organ vascular diversity and arterio‐venous hierarchies complicate the molecular characterization of the vasculature's cellular building blocks. Single‐cell RNA sequencing provides a way forward, as it allows elucidation at a genome‐wide and quantitative level of the transcriptional diversity occurring within the same cell types at different anatomical positions and levels of arterio‐venous hierarchy in the organs. In this *Louis‐Jeantet Prize Winner: Commentary*, I give a brief overview of vascular development and how recent advances in the field pave the way for more systematic efforts to explore vascular functions in health and disease.

A pressurized cardiovascular system is imperative for the transport of oxygen, nutrients, metabolites, and blood cells in organisms throughout the vertebrate kingdom. The vasculature is of fundamental importance for the body's physiological functions, but it is also required for embryonic development. For some organs, development and function are temporally separated, and the embryo may live and continue to grow in situations where the organ is malformed, or even missing. The vasculature, however, must develop and function simultaneously. This requires precisely coordinated cellular behaviors that shape and reshape the vessel tree while at the same time assuming new, and maintaining already established, vascular functions.

The problem is amply illustrated by studies of mouse gene knockout mutants in which embryonic vascular developmental defects cause general circulatory dysfunction followed by rapid death and disintegration of the embryo. The strict dependence of organism development on vascular function makes the study of embryonic vascular development challenging; it is notoriously difficult to distinguish primary effects of the genes and proteins of interest from the secondary systemic consequences of vascular dysfunction. In this perspective, zebrafish has become a valuable complement to mammals as animal models for the study of vascular development, since part of the oxygenation of the fish larvae takes place across the body surface, allowing at least some aspects of the vasculature to develop even in the absence of a functional circulation. The transparency of zebrafish larvae has also made it possible to image vascular development in real time and at high resolution, something that is still technically very challenging in mammals.

Vascular organotypicity—the adaptation and specialization of the microvasculature to match every organ's specific physiological functions—adds a further dimension of complexity to vascular development. Organotypic vascular specialization generates vast differences in functions such as permeability and molecular transport across the vessel wall. Extreme examples of this specialization include the largely impermeable vasculature of the brain—the blood–brain barrier (BBB)—and the highly permeable, yet size‐selective, capillaries of the kidney glomerulus. All organs have their unique microvasculature, reflected in differences in gene expression patterns and functions, but the details of these differences are only beginning to emerge. The development, maintenance, and derangement of vascular organotypicity are gaining increasing attention in cardiovascular medicine. The importance of these processes is underscored by the vascular contributions to disease processes in several organs, including the brain and the kidney, in metabolic disorders and in cancer.

## Endothelial cell development and angiogenic sprouting

Vascular development is commonly divided into *vasculogenesis*—the differentiation of endothelial cells and their assembly into tubular networks, and *angiogenesis*, the subsequent growth, sprouting, and remodeling of the vascular tubes. It might be appropriate to dub “*organotypogenesis”* the third—and final—process in vascular development. All three stages require cell–cell communication, involving numerous different signals, emanating from cell‐bound (juxtacrine), as well as paracrine (secreted, fixed or soluble) or endocrine (blood‐borne) ligands, the most famous of which is vascular endothelial growth factor (VEGF‐A). Intriguingly, VEGF‐A plays a role in all three stages of vascular development. VEGF‐A's effects on endothelial cells are context‐dependent, and the molecule is a clinical drug target in cancer and eye diseases (Ferrara & Alitalo, 1999).

Endothelial progenitors arise in the mesoderm under the influence of fibroblast growth factor and bone morphogenetic protein, and their further coalescence into the first vascular tubes depends on signaling elicited by hedgehog, delta, and VEGF‐A ligands. One of the first markers for the endothelial progenitors Flk1 (VEGF receptor 2) is also the major cell surface receptor for VEGF‐A. However, the process of vascular cell differentiation from undifferentiated mesenchyme probably continues also after a functional circulatory system has formed, and hence, vasculogenic and angiogenic mechanisms are likely not temporally separate processes.

**Figure 1:**
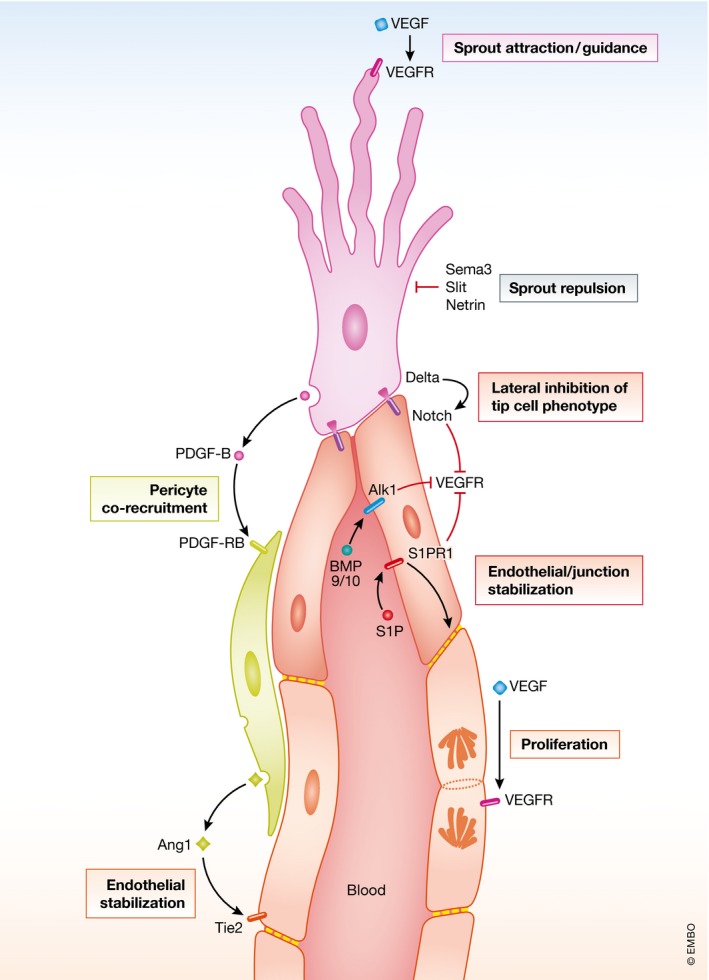
Cell–cell signaling in angiogenic sprouting The figure illustrates ligand–receptor signaling in an angiogenic sprout composed of a single endothelial tip cell, several lumen forming stalk cells and a co‐recruited pericyte. Some of the ligand and receptors are indicated with the family names instead of specific members. The different cellular functions/processes are boxed.

VEGF‐A is equally the major regulator of angiogenic sprouting. VEGF‐A expression is under strong transcriptional control of environmental influences, one of which is hypoxia (Shweiki *et al*, 1992), ensuring that VEGF‐A is produced and vessels are formed where they are needed. The developing retina provides a good illustration to this paradigm. It gets vascularized through centrifugal sprouting from feeding vessels entering from the optic nerve head located at the retinal center. Since the postnatal mouse retina presents an essentially two‐dimensional vascular network with a clear central‐to‐peripheral direction of angiogenic sprouting, and since it displays several distinct angiogenic processes simultaneously, that is, sprouting at the periphery and remodeling into arteries, veins, and capillaries at central locations, it has become a popular model for the study of angiogenesis. It was also the site where endothelial specialization into tip cells and stalk cells, and the differential response of these cells to VEGF‐A—migration of tip cells and proliferation of stalk cells—were uncovered (Gerhardt *et al*, 2003). The tip‐ and stalk‐cell phenotypes are dynamic states upheld through a lateral inhibition mechanism involving Notch signaling. Many layers of regulation and fine‐tuning of these processes have been unraveled, involving the precise adjustment of VEGF‐A levels and signaling intensity via VEGFR2 in tip cells and Delta‐like ligand 4‐mediated activation of Notch1/4 in stalk cells. Moreover, numerous other signaling pathways known from other developmental processes have been implicated in the control of angiogenic sprouting, such as BMPs, and axon guidance molecules of the Ephrin/Eph, Semaphorin/Plexin and Slit/Robo families (Eichmann & Thomas, 2013). The navigation of neurites and endothelial cells through tissues thus appears to involve the same type of molecular cues and morphogenetic principles (Fig 1).

## Recruitment of mural cells to developing vessels

Endothelial cells are not the only vascular cells. A second cell type—the vascular mural cell—is an obligatory component of all blood vessels. Mural cell is the collective term for pericytes and vascular smooth muscle cells (VSMC), which occupy small and large vessels, respectively. Pericytes and VSMC are presumed to be related and to have a common ontogeny. However, the details of mural cell development and phenotypic diversity remain largely unexplored.

During angiogenesis, platelet‐derived growth factor‐B (PDGF‐B) is released from endothelial cells for paracrine signaling through PDGF receptor‐β (PDGF‐Rβ) on pericytes, stimulating their proliferation and recruitment along the angiogenic sprouts (Lindahl *et al*, 1997). This process is conserved between mouse and fish and was recently recorded live in zebrafish larvae (Ando *et al*, 2016). While PDGF‐B/Rβ signaling is clearly fundamental for pericyte recruitment, it is not required for the emergence of mural cells. Other—yet to be elucidated—mechanisms appear to drive the “vasculogenic” aspect of mural cell development. Moreover, while PDGF‐B/Rβ signaling is critical for pericyte recruitment to the vessels in the central nervous system and some other organs, pericytes at other locations, including the liver, are normally recruited or merely quantitatively reduced in the absence of PDGF‐B/Rβ. The nature of the signals involved in PDGF‐B‐independent pericyte recruitment remains unknown.

Without PDGF‐B/Rβ, and hence devoid of pericytes in some but not all organs, mouse embryos survive through late gestation, but invariably die at birth from generalized hemorrhage and edema. Patent (lumenized) vessels form in the absence of pericytes and vascular arterio‐venous hierarchies are established, but the capillaries fail to mature normally. Studies of both knockout and hypomorphic *Pdgfb* mutants have revealed two hallmarks of pericyte‐deficient capillaries: the loss of strict capillary diameter control (variable and generally dilated diameter, as opposed to the strictly uniform 4–5 μm diameter) and increased vascular leakage resulting in edema (Lindahl *et al*, 1997).

## Vascular organotypicity

The molecular basis for vascular organotypogenesis and the cellular crosstalk required for this differentiation remain unknown with a few exception (Augustin & Koh, 2017). Organotypic vascular signaling is reciprocal; that is, the vascular cells are induced to assume organotypic features by molecular cues released from the target organ, but the endothelial cells—and possibly also the pericytes—also release “angiocrine” cues that influence the differentiation of the target organ itself (Rafii *et al*, 2016). This reciprocal cell–cell signaling is thus similar to the epithelial–mesenchymal crosstalk that is central in the development of all epithelial organs.

For one of the best studies vascular organotypic states—the BBB—Wnt signaling has been demonstrated to be essential for BBB differentiation of the endothelium. Intriguingly, Wnt inhibitors released by certain types of medulloblastoma tumors lead to loss of endothelial BBB differentiation (Phoenix *et al*, 2016), showing that the BBB is a plastic organotypic state under the influence of a disease process. Likely, the loss of vascular organotypicity is a hallmark of many other diseases, such as those of the kidney glomerulus causing reduced blood ultrafiltration and eventually chronic renal failure. The signals maintaining or disrupting vascular organotypicity—once known—may therefore turn out to be clinically useful drug targets.

Also endothelial–pericyte signaling may be critical for establishing or maintaining vascular organotypicity. It is possible that pericytes provide organ‐unique cues that regulate functions in the surrounding cells, including the endothelium. In the brain, loss of pericytes affects specific aspects of the BBB, including the activation of endothelial transcytosis resulting in increased bulk fluid transport from blood to brain (Armulik *et al*, 2010). How the pericytes exert this function—that is, what signals they provide and what responses these trigger are currently unknown.

## Challenges and promises ahead

The analysis of cell–cell signaling in vascular development presents several fundamental challenges, one of which is the organotypicity and the need for studying vascular cell–cell signaling at the organ‐ and sub‐organ—rather than at the organism level. Sub‐organ vascular specialization is evident in many, if not all, organs. The kidney, harboring distinct micro‐vascular functions in the glomerulus (ultrafiltration) and tubular systems (re‐absorption), is an obvious example, but most organs likely display micro‐vascular heterogeneity. The brain contains small regions where the BBB is atypical or missing, such as the choroid plexus (involved in CSF production) and the circum‐ventricular organs in which hormonal exchange takes place between the brain and periphery. Arterio‐venous (A‐V) hierarchy is another level of intra‐organ vascular heterogeneity, and it is likely (although the details remain to be elucidated) that vascular functions and associated gene expression patterns vary along the A‐V axis. Thus, isolation of vascular cells in bulk from an organ for molecular profiling will inevitably provide an average, and hence erroneous, picture of the vascular cell phenotype. For example, if our aim is to reveal the nature of cell–cell signaling between capillary endothelial cells of certain specialized vessels and their associated pericytes, or between arterial endothelial cells and their neighboring SMC, it is imperative that the vascular cell subtypes can be separated before analysis. Generally, the appropriate tools (e.g. antibody markers and transgenic reporters) do not exist for such work, although there are exceptions, such as protocols for the separation of glomeruli from the rest of the kidney.

Rather than isolating pure populations—cohorts—of vascular cell subtypes for molecular profiling, recent developments in single‐cell RNA sequencing (scRNASeq) make it possible to isolate cells “at random”, determine their gene expression, and reveal their identity in retrospect through clustering, and their anatomical location using appropriate (combinations of) markers (Zeisel *et al,* 2015). Preliminary insight into vascular single‐cell transcriptomes, such those of the brain, have been obtained as “side findings” in work dedicated to other cell types, but these data are still scant and interpretations hampered by the small size and low RNA content of vascular cells, as compared to e.g. larger epithelial and neuronal cell types. Nevertheless, dedicated efforts aiming at single‐cell profiling of the vasculature are in the works and will likely revolutionize our understanding of vascular diversity and cell–cell signaling in angiogenesis and other aspects of vascular physiology and pathophysiology.

## Conflict of interest

The author declares that he has no conflict of interest.
